# Disreputable

**Published:** 1879-10-20

**Authors:** 


### *DISREPUTABLE.*

The following extract we clip from a newspaper published at Hot Springs, Arkansas :

"DRUMMING.—To-day we republish our article on drumming, with Dr. Adams' petition praying for an injunction to prevent the city authorities interfering with the practice of drumming, roping, steering, or whatever else the public may please to term it. We take this step for the purpose of supplying the demand for papers on the part of the respectable physicians of this place, who wish to send them to members of the medical profession throughout the country, as the best evidence of the infamy practiced daily by the quacks of our city.

"Hundreds of patients come to this city every month with letters to respectable physicians, but drummers run them in to quacks, who rob them of everything but their infirmities; and like the bunko sharps, never refund a cent, not even pretending to render any equivalent when their dupes once find themselves utterly sold, robbed and plundered, and left without means amongst strangers, helpless with their infirmities, and with the quacks, drummers, boarding houses and hotels all in league against them, while the city authorities are to be rendered helpless to give them protection.

"It is the experience of all persons the world over that the quack always pretends to be a regular, honorable and experienced physician, claiming extraordinary skill, etc. The case in question is the first instance within our knowledge where a physician boasts of the means he employs, on the excuse that other equally infamous practices are resorted to by those who can subsidize hotels and boarding houses to accomplish similar results. It will, therefore, be easily seen by the profession at large how their patients are liable to fall into the hands of sharks, who prey upon the infirmities of invalids seeking relief from their sufferings at this wonderful natural resort."

